# Compas-Y: A mixed methods pilot evaluation of a mobile self-compassion training for people with newly diagnosed cancer

**DOI:** 10.1177/20552076231205272

**Published:** 2023-10-19

**Authors:** Judith Austin, Maya J Schroevers, Jelle Van Dijk, Robbert Sanderman, Elin Børøsund, A Machteld N Wymenga, Ernst T Bohlmeijer, Constance H.C. Drossaert

**Affiliations:** 1 Section of Psychology, Health and Technology, 3230 University of Twente, Enschede, The Netherlands; 2 Department of Health Psychology, University Medical Center Groningen, University of Groningen, Groningen, The Netherlands; 3 Faculty of Engineering Technology, 3230 University of Twente, Enschede, The Netherlands; 4 Department of Digital Health Research, Division of Medicine, 155272 Oslo University Hospital, Oslo, Norway; 5 Department of Internal Medicine, Medisch Spectrum Twente, Enschede, The Netherlands

**Keywords:** Self-compassion, cancer, eHealth, mental well-being, mixed methods, compassionate mind training

## Abstract

**Objective:**

Compas-Y is a compassionate mind training app that was co-designed to be fully adapted to mobile technology and to people with newly diagnosed cancer. This study aimed to evaluate the use, appreciation and impact of the app.

**Methods:**

Seventy-one people with cancer who created an app account were included (38% breast cancer, 72% diagnosed <4 months ago, 76% received chemotherapy). Participants had very high baseline scores of self-compassion. In a convergent mixed methods design, back-end log-data (*n *= 71), pre-post surveys (*n *= 34) and semi-structured interviews (*n *= 23) collected for >8 weeks and were concurrently analysed using joint displays.

**Results:**

About half of the participants (45%) used 4 of the 6 modules. Compas-Y was highly appreciated, with all content considered relevant and a source of support. Experienced benefits related to improved mental health. Particularly, we found significant changes in anxiety, but not in depression or well-being. In the interviews, people reported experiencing more rest and more positive emotions due to using the app. Process benefits included significant reductions in self-criticism (inadequate self and self-blame), but not self-compassion. In the interviews, people reported improved self-compassion and less self-criticism, more self-awareness, recognition and support, and improved emotion regulation and coping. The surveys did not capture the full range of outcomes that participants reported in the interviews.

**Conclusions:**

Compas-Y is a highly appreciated mobile intervention that supported users in aspects of their mental health. Findings are discussed in terms of reach and adherence, app functionalities, co-design and tailoring of cancer-related and compassion-based eHealth.

## Introduction

eHealth interventions for people with cancer aimed at supporting well-being are increasingly being developed and investigated.^1^ These interventions usually contain a mix of informative and interactive elements, including psychoeducation, self-monitoring, and exercises.^2^ Offered as a stand-alone intervention or as an addition to face-to-face interventions, eHealth may provide unique benefits related to increased accessibility and scalability^3^ and integration of new skills into daily life.^4^ While eHealth can support people with cancer in all stages of the illness trajectory,^1^ the majority of current eHealth interventions focus on people who have completed treatment.^5^ Particularly for people with newly diagnosed cancer who still receive medical treatment, eHealth may offer a low-threshold form of support that many may find more feasible than traveling to and attending face-to-face sessions.^6^

Having cancer and receiving treatments such as chemotherapy often cause severe physical symptoms such as nausea and fatigue.^7,8^ In addition, people with cancer are at an increased risk of anxiety and depression,^9,10^ and may experience problems in their social roles and activities.^8^ Moreover, many people with cancer tend to be self-critical with regard to how they manage these consequences of the illness and its treatment.^11^ This may be in the form of being harsh with oneself by setting high standards of productivity comparable to pre-diagnosis, feeling useless for not being able to perform social roles, or feeling guilty for unhealthy behaviours.^12^ In contrast to self-criticism, self-compassion is a way to be sensitive to the suffering in oneself, with a commitment to try to alleviate and prevent it.^13^ In people with cancer, self-compassion has been linked to lower distress^14–16^ and higher resilience.^17^

Compassionate mind training (CMT) is an intervention to cultivate (self-)compassion and reduce resistance to (self-)compassion. Central in the training is a model of three evolved emotion systems, that enable gathering resources (drive system), detecting and protecting against threats (threat system) and regenerating and affiliating with others (soothing system).^13^ For people with cancer, the threat system may be overactive, often described as being in ‘survival mode’.^12,18^ Although the drive system is primarily one of positive affect (e.g. excitement), an overreliance on achievement or acquiring can lead people to feel exhausted and fatigued.^13^ For people with cancer this may present as a strong focus on acquiring as much (health) information as possible, despite adverse effects.^18^ In CMT, experiential, meditative and reflective exercises are offered to balance these emotion systems and to foster compassionate qualities and skills, including awareness of ways in which people tend to be self-critical.^19,20^

Compassion-based interventions such as CMT contribute to an improved mental health in people with long-term physical conditions, for example by decreasing anxiety or depression and improving emotion regulation.^21^ However, existing compassion-based interventions mostly take place face-to-face with minimal use of technology, and are also not tailored to people with cancer (see review^21^). The handful of compassion-based eHealth interventions found in literature are directed at non-clinical populations and are mostly offered in a web-based, pdf or e-mail format,^22–25^ with the exception of a mobile intervention for adolescents that offers exercises via push notifications.^26,27^ Therefore, it is still unclear how benefits of (mobile) technology such as tailoring, personalisation and notifications^28^ can be leveraged for compassion-based interventions. Comprehensive evaluation of such eHealth interventions, including not only effectiveness but also use and appreciation, is needed to shed light on this.^29^ Furthermore, it is important to evaluate not only interventions as a whole but also the components they consist of, since people tend to use the same eHealth technology in very different ways (i.e. using some components but not others).^29^

This mixed methods pilot study therefore aimed to evaluate the use, appreciation and impact of the newly developed mobile self-compassion training *Compas-Y*. *Compas-Y* was co-designed based on CMT theory and evidence as well as experiences, wishes and needs of people with cancer, with the aim of offering a tailored and low-threshold form of support after receiving a cancer diagnosis. An elaborate description of the co-design process can be found elsewhere.^18^ Given the complex context of (eHealth) intervention research, mixed methods are particularly suited to evaluate intervention processes and outcomes.^29–33^ Mixed methods research involves a synthesis of different strands of data, typically quantitative and qualitative, which are integrated during all phases of a study.^34^ When this includes mixed analysis, meta-inferences can be drawn that facilitate comprehensive evaluation of app use, appreciation and impact.^32,35^ The results of this study are expected to contribute not only to the evidence-base of this particular intervention, but also of cancer-related and compassion-based eHealth in general.

## Methods

### Study design

This study employed mixed methods to assess the use, appreciation and impact of a mobile self-compassion intervention for people with newly diagnosed cancer (*Compas-Y*). Mixed methods were chosen for reasons of complementarity,^36^ that is, to build a deeper and more complete understanding of the intervention. In this convergent design, pre-post surveys, back-end log-data and semi-structured interviews were collected simultaneously and with equal priority given to the different data strands.^34^ In co-design terms, this study is part of post-design, that is, the evaluation of the app in a daily life context which may prompt further design decisions.^37^

### Intervention

**Figure 1.**
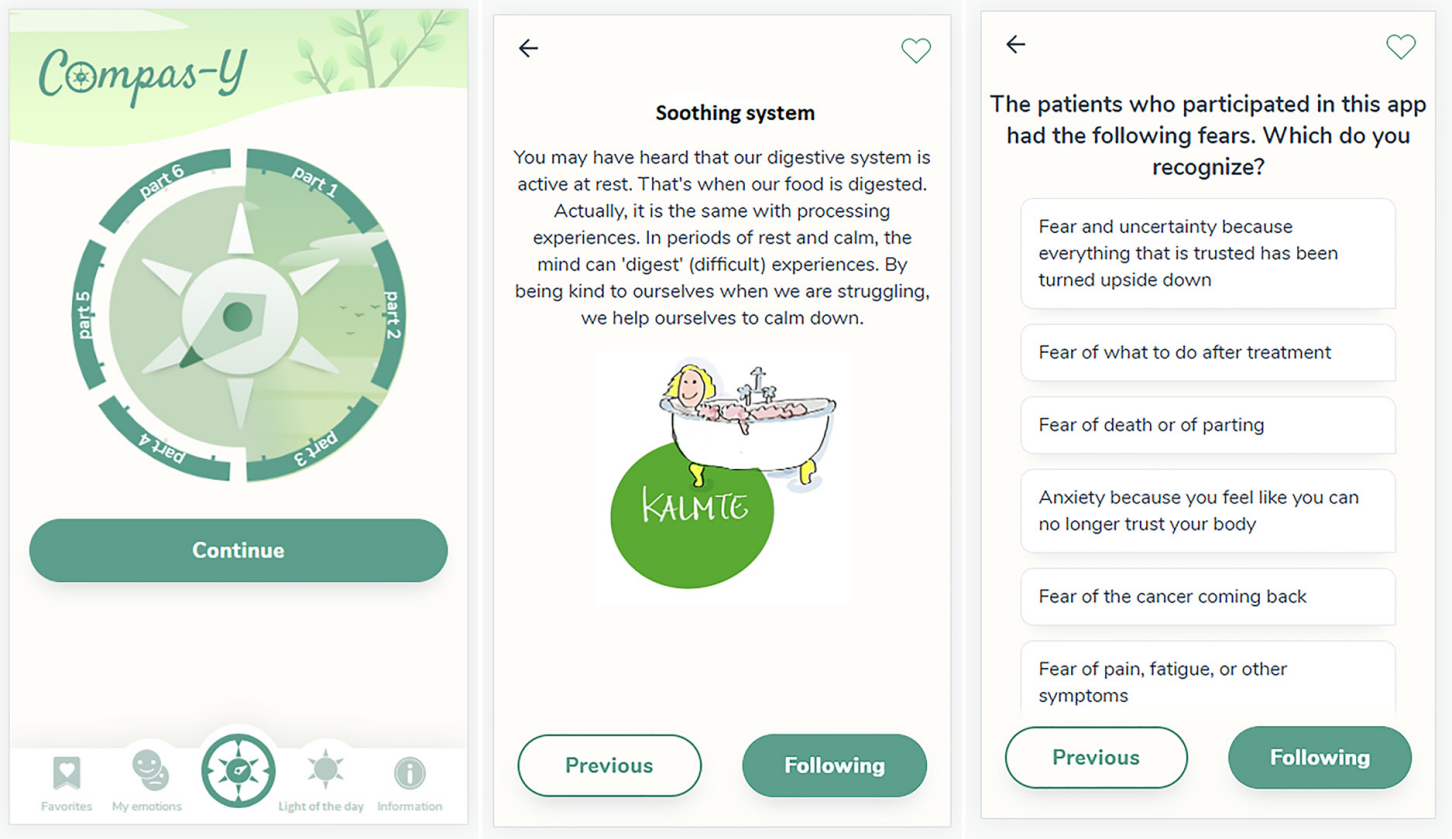
Screenshots of Compas-Y (auto-translated). App homepage (left); psycho-education about taking rest as a way to process (difficult) experiences, after which the user will be invited to build in moments of rest (middle; soothing system exercise); multiple choice reflection question about recognising possible sources of anxiety in relation to the diagnosis (right; threat system).

*Compas-Y* represents a version of CMT tailored to the context of cancer and to the modality of a mobile application, which was co-designed together with people with cancer and oncology nurses (see Ref.^18^ for the co-design process and full intervention details including a video demonstration). Table 1 presents an overview of intervention content. Essential components of CMT include psycho-education and practices about resistance to and motivation for self-compassion, the model of the three emotion systems and exercises such as soothing rhythm breathing and imagery.^19,20^ For instance, by cultivating affiliative emotions (soothing system) with compassion exercises, the capacity to engage with threat-based emotions such as anxiety is increased. Participants may be encouraged to build in moments of rest in de midst of turbulent times, and to recognise and regulate anxieties related to their diagnosis (see Figure 1 for example screenshots). The six sequential training modules each have a central theme, in which one or more topics proposed by the co-designers are intertwined with content from CMT (e.g. anxieties related to treatment outcomes are addressed in the context of the threat emotion system). Modules consist of psycho-education, audio-guided, experiential and reflective exercises and peer experiences. Peer experiences are marked as optional (since co-designers tended to find these either essential or unwanted/confronting). Modules are delivered in a mixture of brief textual and audiovisual format (43–53 screens; estimated 60–90 minutes to complete). Modules open after 1 week regardless of completion of prior modules and participants are notified by push notification. Modules can be revisited at any time. In addition, supportive functionalities that are directly accessible from the homepage include a page with favourite exercises, a mood tracker based on the three emotion systems, an exercise in which the user reflects on a pleasant experience (light of the day) and a practical information page. Brief exercises and inspirational quotes are sent up to once a day via push notifications. Other persuasive design elements include automated feedback, progress tracking and visual rewards (see Refs.^18,28^).

**Table 1.** Overview of app modules and functionalities with their key components.

Module	Key components and exercises
1. Introduction to the app and self-compassion	Psycho-education about self-compassion Exercises in mindful awareness and soothing breathing rhythm Exercise in finding small positive moments during the day
2. Emotions in the context of cancer	Psycho-education about three emotion systems (soothing, drive and threat) Soothing breathing rhythm exercise with imagery (soothing) Compassionate information seeking; finding resources based on own needs (drive) Psycho-education about anxiety; practicing to recognise and allow anxiety (threat)
3. Self-compassion and self-criticism	Psycho-education about self-compassion and self-criticism Imagery exercises about compassionate self and inner critic Soothing breathing exercise with compassionate friend Self-compassion expressive writing exercise
4. Taking care of your body	Soothing breathing rhythm-based compassionate body scan Psycho-education and exercises about compassionate motivation and self-correction vs. self-critical motivation or attacking related to lifestyle behaviours Psycho-education about compassion for own needs in the context of sexuality and intimacy
5. The people around you	Psycho-education about the three flows of compassion Soothing breathing rhythm-based loving-kindness meditation Setting boundaries and asking for help based on compassion for own needs
6. Continuing with resilience	Psycho-education and exercises about positivity as a resource of resilience: gratitude, savoring, strengths Reflection on self-compassion practice and how to continue Soothing breathing rhythm meditation with focus on tone of voice, posture etc.
*Supportive functionalities*	*Description*
Overview of modules	Visual element central to the homepage (compass symbol) that depicts the (availability of) 6 modules and user progress
Mood tracker	Mood tracking (one slider question for each emotion system) with automated feedback based on three emotion systems. ‘*Example feedback: Your threat- and drive system are active. Perhaps you’re feeling rushed, angry or anxious. Calm seems far away. What helped you in the past to experience rest? If you’re having trouble thinking of something, you could try some of the exercises below.*’
Favourite exercises	Marking any page or exercise as favourite within the modules, which then appear in the users’ personal list of favourites
Light of the day	Exercise where user types a positive moment of their day, supported by examples
Practical information	List with weblinks about (living with) cancer, each with descriptions
Push notifications	Daily messages containing quotes and brief exercises, with an option to reduce the frequency or turn messages off. *Example message: ‘You can be your own supporter instead of opponent. What wise encouragement or compliment can you give yourself today?’*

Reproduced and adapted under the terms of Creative Commons Attribution 4.0 license.

### Participants and procedure

**Figure 2.**
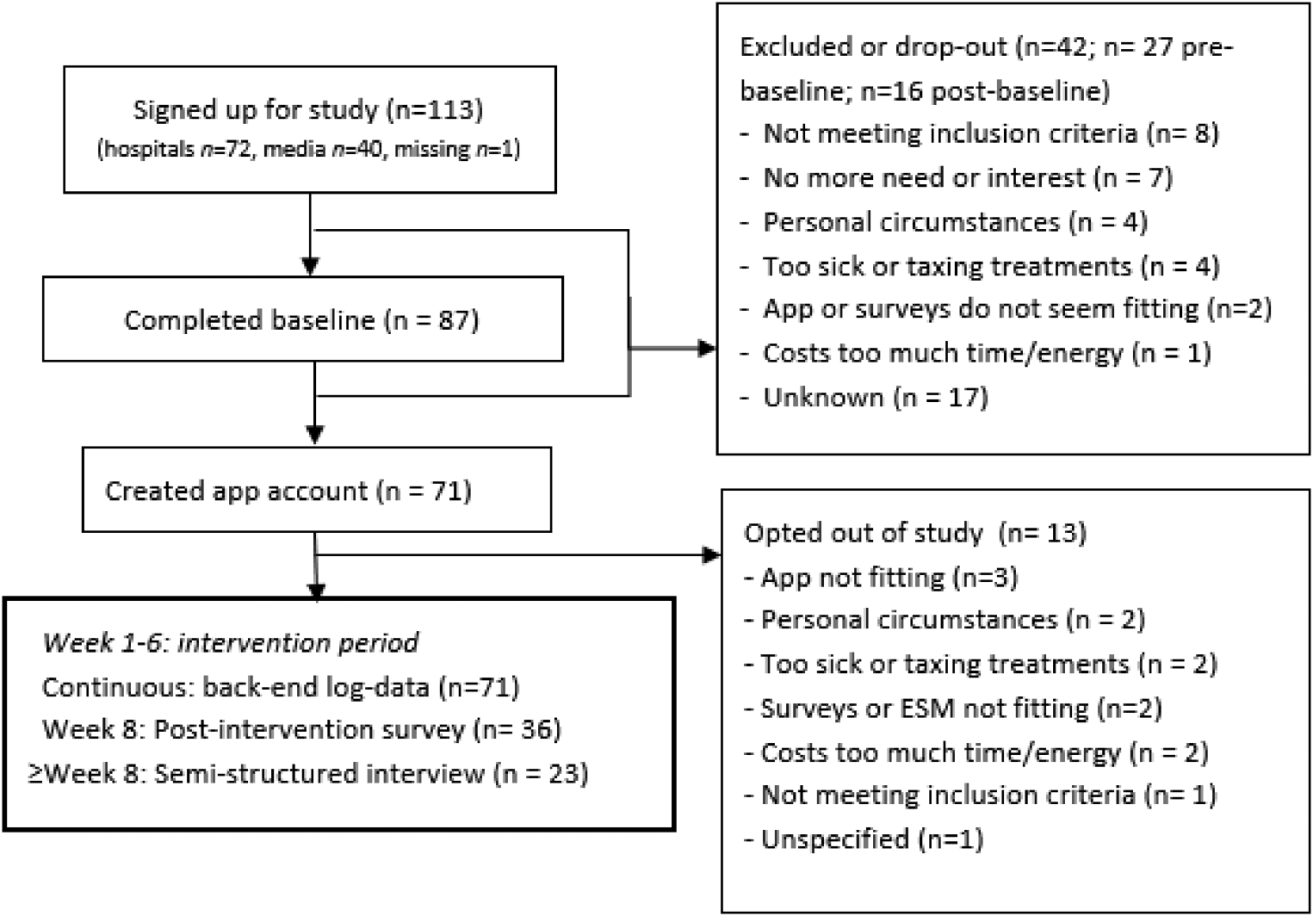
Participant and study flow.

Within two hospitals, participating oncology nurses attempted to recruit all adults (≥18 years) with cancer who received any diagnosis of cancer < 12 months ago and who received treatment with curative intent (i.e. purposive criterion sampling^38^). The two hospitals were a community hospital in the East and a university hospital in the North of The Netherlands, selected for practical reasons (i.e. previously established collaborations) as well as to achieve broader representation of variable hospital contexts. During a standard care consultation shortly after diagnosis, oncology nurses gave eligible people with cancer a leaflet with information about the intervention, the research context and a weblink for more information and sign-up. Due to COVID-19-related recruitment delays, the app was later also offered online and promoted via cancer-related and general media outlets. Following sign-up, participants received a link to the informed consent and baseline questionnaire, and upon completion an access code for the intervention was automatically sent. Participants filled out outcome measures online at baseline and 8-week post-intervention taking 2× 15–25 minutes to complete. In week 3 of the intervention, an experience sampling methods (ESM) study was conducted that is not part of the current article. Participants filled out questions addressing self-kindness, self-judgment and mood (four times a day, 30 seconds estimated completion time). At 1 week after sign-up, participants were contacted by telephone by a researcher to inquire about any questions and about starting up (e.g. ‘*Did you manage to add the app to your home screen?*’). See Figure 2 for the participant and study flow.

### Measures

#### Pre-post surveys

*Sociodemographic and illness variables* were collected with multiple choice and open-ended questions at baseline. Regarding illness information, type and timing of diagnosis and current/recent primary and additional treatment were assessed. Regarding demographic information, age, gender, marital status, cultural identification, employment and education were assessed. Furthermore, prior experience with mindfulness and self-compassion training or exercises, type and frequency of smartphone and computer use and motivation for participating in the study were assessed.

*App evaluation* was assessed with multiple choice and open-ended questions at post-intervention (8 weeks). For app use, time spent on the app per week and use of separate modules and supportive functionalities were assessed. For app appreciation, respondents rated module content, supportive functionalities, and form aspects such as visual design and language. They also described their three favourite app components and were asked for suggestions for improvement. For impact, respondents described what the app brought them and in what ways it was (un)supportive. In addition, the TWente Engagement with Ehealth Technologies Scale (TWEETS)^39^ was used for app evaluation. It measures past engagement (behavioural, cognitive and affective engagement) with the intervention on a 9-item scale at post-intervention, and includes indicators for use (e.g. ‘[*Compas-Y*] became part of my daily routine’), appreciation (e.g. ‘[*Compas-Y*] was easy to use’) and process outcomes (e.g. ‘[*Compas-Y*] made it easier to be self-compassionate’). Scoring for each item ranges from 0 *strongly disagree* to 4 *strongly agree*. In the current sample, the scale showed good reliability (*α* = .88 at post-intervention).

#### Mental health outcomes

*Anxiety and depression* were assessed with the Hospital Anxiety and Depression Scale (HADS)^40^ on a 14-item scale. Respondents rate the extent to which they have been feeling anxious (e.g. ‘I feel tensed lately’) or depressed (e.g. ‘I feel cheerful’) on 2 subscales of 7 items each. Scoring for each item ranges from 0, for example, *most of the time* to 3, for example, *not at all*, with 3 indicating the highest anxiety or depression (after reverse scoring some items). A total subscore of >8 indicates considerable anxiety or depression. In the current sample, the scale showed good reliability (HADS-A *α* = .87 and *α* = .86; HADS-D *α* = .84 and *α* = .79 at pre- and post-intervention, respectively).

*Well-being* was assessed with the 14-item Mental Health Continuum Short Form (MHC-SF).^41^ Respondents rate the extent to which they have been experiencing emotional, social, and psychological well-being (e.g. ‘In the past month, how often did you feel confident to think or express your own ideas and opinions?’). Scoring for each item ranges from 0 *never* to 5 *every day*, with 5 indicating the highest level of well-being. In the current sample, the total scale showed good reliability (*α* = .91 and *α* = .92 at pre- and post-intervention, respectively).

*Social isolation* was assessed with the Patient-Reported Outcomes Measurement Information System (PROMIS)-social isolation^42^ on an 8-item scale. Respondents rate the extent to which they recently have been feeling left out, isolated, and detached from others (e.g. ‘I felt isolated from others’). Of note, the study period coincided with the COVID-19 pandemic which provided notable and variable measures of social isolation. Scoring for each item ranges from 1 *never* to 5 *always*, with 5 indicating the greatest perceived social isolation. In the current sample, the scale showed good reliability (*α* = .90 and *α* = .89 at pre- and post-intervention, respectively).

*Cancer-related resilience* was assessed with an adapted version of the Brief Resilience Scale (BRS)^43^ on a 6-item scale. Following feedback of patient advocates to include more cancer-related questions in the study, we adapted the BRS to focus on the context of cancer (Brief Cancer-related Resilience Scale; BCRS, see Supplementary File 1). Respondents rate the extent to which they tend to bounce back after cancer-related challenges (e.g. ‘I tend to bounce back quickly after a difficult phase of my illness or treatment’ and ‘I need a long time to get over the fact that I have cancer’). Scoring for each item ranges from 1 *strongly disagree* to 5 *strongly agree*, with 5 indicating the greatest resilience (after reverse scoring some items). In the current sample, the adapted scale showed good reliability (*α* = .79 and *α* = .81 at pre- and post-intervention, respectively).

#### Process outcomes

*Self-compassion* was assessed with *the Self-compassion Short Form* (SCS-SF)^44^ on a 12-item scale. Respondents indicate the extent to which they usually experience aspects of self-compassion including self-kindness and self-judgment, common humanity and isolation, mindfulness, and over-identification (e.g. ‘When I fail at something important to me, I become consumed by feelings of inadequacy’). Scoring on the Dutch scale ranges from 0 *seldom or never* to 7 *almost always*, with 7 indicating the highest frequency of experiencing self-compassion. Recently, Neff's self-compassion scales have been subjected to much debate regarding the use of the total score and/or subscales (see Refs.^44,45^). Given that we only used the short-form, we focused on the total score, which showed good reliability in the current sample (*α* = .89 and *α* = .87 at pre- and post-intervention, respectively).

*Self-criticism* was assessed with the Forms of Self-Criticising/Attacking and Self-Reassuring Scale Short Form (FSCSRS-SF)^46^ on a 14-item scale. Respondents rate their sense of inadequacy (e.g. ‘I think I deserve my self-criticism’, 6 items), sense of self-hate (e.g. ‘I stop caring about myself’, 3 items) and of self-reassurance (e.g. ‘I am able to remind myself of positive things about myself’, 5 items). Scoring for each item ranges from 0 *not at all like* me to 4 *extremely like me*, with 4 indicating greater self-criticism or self-reassurance. In the current sample, the subscales inadequate self and reassured self showed good reliability (FSCSRS-SF-IS *α* = .73 and *α* = .77, FSCRS-SF-RS *α* = .81 and *α* = .68 at pre- and post-intervention, respectively), while hated-self did not (FSCRS-SF-HS *α* = .42 and *α* = .27 at pre- and post-intervention, respectively). Indeed, the hated-self items seem most applicable to clinical mental health samples^47^ and the scores of this sample were so low as to suggest floor effects. Hated-self was therefore not analysed further.

*Fears of giving and receiving compassion* were assessed with two abbreviated subscales of the Fears of Compassion Scales (FOCS).^48^ The original subscales have 10 and 13 items, respectively, and were shortened to 4 items for reasons of feasibility. Respondents rate the extent to which they agree with thoughts and beliefs about expressing compassion for others (e.g. ‘people need to help themselves rather than waiting for others to help them’), and fear of receiving compassion from others (e.g. ‘I often wonder whether displays of warmth and kindness from others are genuine’). Scoring for each item ranges from 0 *don’t agree at all* to 4 *completely agree*, with 4 indicating the greatest fears of compassion. In the current sample, the abbreviated subscales showed okay (FOCS-giving *α* = .61 and *α* = .66) to good reliability (FOCS-receiving *α* = .82 and *α* = .76) at pre- and post-intervention, respectively, albeit lower than for the full subscales (FOCS-giving *α* = .84; FOCS-receiving *α* = .85^48^).

*Cognitive coping and emotion regulation strategies* were assessed with the Cognitive Emotion Regulation Questionnaire Short Form (CERQ-SF)^49^ on an 18-item scale. Respondents rate the extent to which they use nine different strategies: self-blame, acceptance, rumination, positive refocusing, planning, positive reappraisal, putting into perspective, catastrophising, and other-blame. These subscales each have two items and scoring for each item ranges from 0 *almost never* to 4 *almost always*, with 4 representing the highest use of a strategy. In the current sample, the subscales predominantly showed predominantly good reliability (*α* = .64–.93 and *α* = .49–.96 at pre- and post-intervention, respectively).

#### Log-data

Back-end log-data were automatically collected whenever a participant created an app account, as indicated in the informed consent and privacy statement. Logged were any in-app actions (e.g. opening a module, adding a light of the day) in conjunction with a time stamp and an e-mail identifier throughout the study period (e.g. participant@testmail.com listened to audio-guided exercise no. 4 at 23-03-2022 14.03 hours). For privacy reasons, in-app input (e.g. answers to open or multiple choice questions) was not recorded.

#### Interviews

In the 8-week post-intervention questionnaire, participants were asked to indicate their interest for participating in a semi-structured interview (‘yes’, ‘perhaps’ or ‘no’), and were then contacted to set up a meeting (i.e. convenience subsample). All participants who created an app account were asked to do an interview unless they opted out of the study (see Figure 2). Throughout study materials it was emphasized that all kinds of experiences would be welcomed in the interviews, including negative experiences or experiences after limited app use. Interviews were conducted by a researcher and two student assistants with prior interviewing experience. Interviews took place via videoconferencing and lasted 20–60 minutes (based on the participants’ energy level) and were transcribed a verbatim.

#### Interview guide

The interview guide consisted of an introduction of the interviewer and the study aims and four parts addressing app evaluation. During the first part, general impressions of the app and most or least helpful, pleasant or valuable aspects were addressed (e.g. ‘*What is your overall experience of using the app?*’). General use, including time spent on the app and what components were used or skipped, was also discussed. In the second part, the six training modules were addressed. This included general impressions and use of the six modules and their coherence (e.g. ‘*To what extent did you gain or learn something by using this module?*’). For each module, the use, appreciation and impact (experienced benefits and potential adverse effects) were discussed separately. In addition, appreciation of audio and video content, peer experiences, receiving feedback, app design and app navigation were highlighted (e.g. ‘*What was it like to read peer experiences?*’). In the third part, the participant was asked about use, appreciation and impact of the supportive functionalities (mood tracker, light of the day, my favourites, information page and push notifications). In the fourth part, the participant was asked about whether they would recommend the app and why (not), and any suggestions for further development of the intervention (e.g. ‘*If there were to be a Compas-Y 2.0, what should be changed and what should be retained?*). If the participant indicated a need for a shorter interview, the second and third part of the interview were shortened based on the participants’ use of app components.

### Data analysis

*The analysis framework* constituted a concurrent approach, with initial separative analysis of the data strands. Equal priority was given to qualitative and quantitative data and each was allowed to inform the points of merging.^50^

*Quantitative analyses* were performed in SPSS version 28.0 and Microsoft Excel version 2208. Descriptive analyses outlined sociodemographic, prior knowledge of self-compassion and illness characteristics as well as app use and appreciation (total appreciation was calculated by summing up all appreciation items). Paired sample *t*-test and Chi-squared analyses were used to examine changes in mental health and process outcomes from pre- to post-intervention (including only participants who completed both time points). Differences between app users and non-users and between interviewees and non-interviewees were compared with independent sample *t*-tests. Significance was set at an *α* of .05 for all tests and Cohen's *d* was calculated to estimate effect sizes. Log-data were analysed using basic frequency analysis, that is, counting the number of views and actions within different app components.^66^ Sum scores were calculated for all different app components (e.g. number of modules used, number of audio-guided exercises played). Pearson correlations between sum scores of app use and T0-T1 change-scores in anxiety, depression, well-being, self-compassion and self-criticism were then calculated to assess dose–response relationships.

*Qualitative analyses* of interview data and answers to open survey questions were performed in Atlas.Ti version 22 and Microsoft Word version 2208. Data were analysed using inductive thematic analysis.^51^ For data familiarisation, interview transcripts were read by one researcher (JA) and two subsets of about 25% and 10% of transcripts was read by a second (CD) and third (MS) researcher, respectively. Next, the first researcher (JA) selected text fragments that were relevant to the research questions which were checked for completeness by the second researcher (CD). The first and second researcher (JA, CD) then independently and inductively sorted text fragments and generated codes, which were sorted into overarching themes (impact). For app use and appreciation, this was only done by the first researcher (JA) given the straightforwardness of the data. All of the results were discussed with two researchers (JA, CD) initially and then with a third researcher (MS). Differences were solved by discussion until consensus was reached.

*Mixed analysis* was performed using joint display analysis for formative data integration.^52–54^ Side-by-side joint displays were created with columns for qualitative findings and quantitative findings, with app components/features (use and appreciation) and group-level impact as the units of analysis. Row-by-row meta-inferences were then formulated, investigating convergence and divergence and iteratively establishing mixed themes. After analyses were performed by the first researcher (JA), points of convergence and divergence and meta-inferences were discussed with the research team (JA, CD, MS, EB) until consensus was reached. A summative figure was then created by ranking app components according to use and appreciation, based primarily on log-data (use) and evaluation scores (appreciation) and supplemented with qualitative and self-report data to differentiate between similar scores. A-posteriori cross-over analyses such as statistics-by-theme joint displays^35,53,55^ were planned if applicable, but were not warranted for the current study (e.g. insufficiently clear thematic differences between interviewees).

## Results

### Study sample

Seventy-one people with cancer created an app account, of which 76% were female and had a mean age of 52 years (range 22–68 years). The most frequently reported reason for participating in the intervention study was a need for support for yourself (*n *= 45 answers), while many also mentioned wanting to contribute to science or better healthcare by sharing their experiences (*n *= 28 answers). Most participants described their cultural identification as Dutch (94%) and were in a committed relationship (81%). About half of participants were engaged in paid work (52%) and completed theoretical education (i.e. (applied) university) (49%). Most participants were diagnosed less than 4 months ago (72%) with breast cancer (38%), and received chemotherapy currently or recently (76%). Participants had higher levels of self-compassion compared to samples of people with cancer and students (see Supplementary File 2 for comparisons of outcome variables with norm scores). Most participants (74–78%) used smartphones, laptops and computers very often for keeping in touch with others and looking up practical information, and least often for tracking their health. There were no differences on any sociodemographic, illness variables or baseline variables between the 23 interviewees and 48 non-interviewees and between the 34 people who filled in pre-post surveys and the 37 people who did not fill in the post-survey. Table 2 shows a full overview of participant characteristics.

**Table 2.** Demographic and illness characteristics participants who created an app account (*n* = 71).

Item	Category	*n*	%	Item	Category	*n*	%
*Age*	*52.5 (range 22–68, SD 11.7)*						
*Gender*	Female	54	76.1	*Time since the diagnosis*	0 < 2 months	27	38.0
	Male	15	21.1		2 < 4 months	24	33.8
	Self-described	0	0		4 < 6 months	6	8.5
	(Missing)	2	2.8		6 < 9 months	6	8.5
*Cultural identification*	Dutch	67	94.4		9 < 12 months	4	5.6
	Mixed or other	4	5.6		(Missing)	4	5.6
*Completed education*	Primary school or none	1	1.4	*Medical treatment(s)*	Chemotherapy	54	76.1
	High school/basic vocational training	17	23.9		Operation	28	39.4
	Vocational education	16	22.5		Immunotherapy	20	28.2
	Theoretical education	35	49.3		Radiotherapy	18	25.4
	(Missing)	2	2.8		Hormonal therapy	11	15.5
*Marital status*	Married/civil partnership/cohabitating	56	81.2		Targeted therapy	5	7.0
	Single/divorced/widowed	13	18.8		None	2	2.8
	(Missing)	2	2.8		Other	6	8.5
*Employment status*	Paid work (employed or entrepreneur)	37	52.1	*Additional treatment(s)*	Physiotherapy	19	26.8
	Sickness benefit (incl. reintegration)	15	21.1		Psychotherapy	10	11.3
	Retired	8	11.3		Acupuncture or alternative medicine	7	9.9
	Homemaker	5	7.0		Revalidation	4	5.6
	Exempted from work	2	2.8		Dietary consultation	4	5.6
	Looking for work, unemployed	1	2.3		Social work	3	4.2
	Other	1	2.3		Other	2	2.8
	(Missing)	2	2.8		None	35	47.9
*Diagnosis*	Breast cancer	27	38.0	*Prior knowledge compassion or mindfulness*	None	31	43.7
	Colorectal or oesophagus cancer	8	5.6		Familiar with it, but not practiced	15	21.1
	Prostate cancer	5	7.0		Followed formal training	10	14.1
	Lymphoma	5	7.0		Did some exercises	7	9.9
	Melanoma	5	7.0		Other	6	8.5
	Ovarium cancer	4	5.6		(Missing)	2	2.8
	Lung cancer	2	2.8				
	Other (*n *= 1 each)	15	21.1				

### Use and appreciation

#### Overall app use

Most participants who completed baseline created an app account (82%, 71/87). No differences were found between these 71 users and 16 non-users on any demographic, illness, or mental health and process outcome variables. Higher total app use was significantly higher amongst participants who also completed post-test surveys (*t *= -3.43(65), *p *= .001). Noteworthy, people who had a higher logged app use (but not self-reported time spent on the app) were more likely to already be familiar with self-compassion (*r*(67) = .30, *p *= .02). Reasons for opening of the app included having a need for information or support, or new content becoming available. Participants described making the app part of their routine. Noteworthy, participants reported that they engaged with the app content also when not using the app (e.g. ongoing reflection). Log-data showed that 69 of the 71 participants (97%) used module one, 47 participants (66%) used module two, 33 participants (47%) used module three, 32 participants (45%) used module four, 25 participants (35%) used module five and 19 participants (27%) used module six, showing a large drop in module use (see Table 1 for an overview of module topics). Qualitative themes for diminished or non-use of the app include feeling too ill or too involved with undergoing treatments, not wanting to be confronted with illness (*‘it's also a kind of fear of negative information’* [D045]) and forgetting to use the app. Interestingly, other reasons included having a low support need (e.g. already having gained sufficient support from the app, ‘*I already got from it what I needed’[D053])* and being mild to yourself by not having to complete everything in the app. See also Figure 2 for the most frequent reasons to disengage with the study and app.

#### Overall app appreciation

##### Appreciation of form: usability, design and presentation of information

Overall, the app was reported to have good to very good usability, design and presentation of information. On the engagement scale, 82% (28/34) (totally) agreed with that the app is ‘easy to use’ (*n *= 34). Qualitative data confirmed the app's simplicity, clarity and usability (*‘it's very easy to use’ [D040]*), its easy navigation as well as clear structure and coherence (‘*the structure makes sense logically*’ [D070]). Related to tunnelling (i.e. structured direction through the app), some found it very helpful to get dosed new content (*‘it was good (…) that it opened up each week (…) that the influx of information was limited*’ [D045]), which could be something to look forward to. The option to skip exercises was appreciated (‘*it's great because you can skip, so I don’t have to do anything with [an undesired component]*’ [D073]). Yet others would have appreciated more flexibility to repeat and skip ahead (‘*it's a downside that I can’t scroll through everything, that I can’t have a look at what I need in that moment*’ [D004]). Most participants (82%; 28/34) rated app design as good or very good, and participants described it as beautiful and calming (‘*it looks good, the colors and sense of calm that it has*’ [D073]). The language use and amount of text were rated as good or very good by 77% (26/34) and 91% (31/34) of participants, respectively. Particularly valued was the mix between the information modalities (audio/video/text/image) and the choices therein. According to participants this made it easier to concentrate and use of the app more enjoyable (‘*when it says, you can also opt to read it [instead of listen], then it immediately feels less coercive*’ [D090]).

##### Appreciation of content: themes and topics addressed in the intervention

Participants found the content of the app pleasant (*‘I find the app fantastic*’ [D028]) and appropriate in the context of a recent cancer diagnosis (‘*it fits immediately when you hear that you have cancer, then it's a great source of information and also a source of hope*’ [D022]). No differences on demographic and illness characteristics (e.g. gender, education, type of diagnosis) were found between people with low or high total appreciation for the app (including both content and form), except for that people who had a higher total appreciation of the app were more likely to already have some familiarity with self-compassion (*r*(34) = .57, *p *< .001). For some people, the content was not completely fitting, as 15% (5/34) (totally) disagreed with the engagement scale item ‘the app fitted me as a person’. Qualitative themes predominantly showed that self-compassion and self-criticism were considered important in the context of cancer (‘*I think that everyone can benefit from it [self-compassion], and that it's extra important in this context. Because you really need self-compassion, and you’re the only one who knows what you need in such a process*’ [D060]). Moreover, all main themes in the app were appreciated in the illness context: the focus on positivity (‘*it gives a lot of positive information, and that's what you need*’ [D022]) and on self-reflection and emotion regulation (‘*if you’re more aware [of emotions/emotion systems] than I think you’re more equipped to cope with such experiences*’ [D093]). When asked for suggestions for improvement, the most supported theme by far is ‘leave the app as it is’.

### Use and appreciation of app components

**Figure 3.**
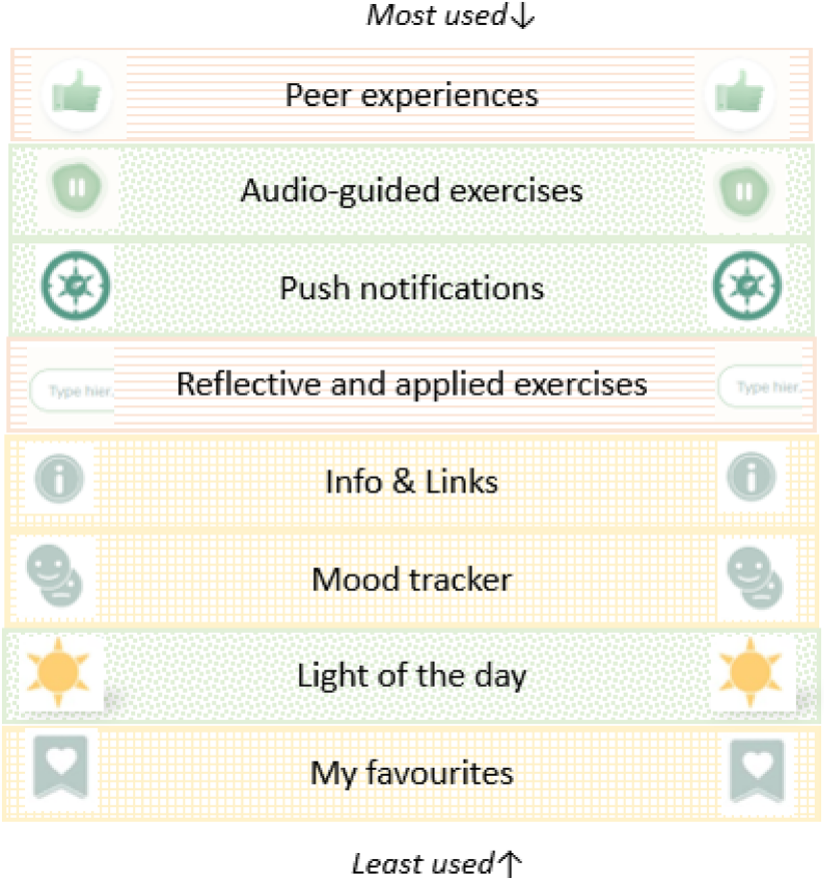
App components ranked by relative use and relative appreciation based on mixed analysis. App components are ranked from most to least used and colors/patterns indicate relative appreciation. Green/dotted pattern = strongest appreciation; orange/striped pattern = medium appreciation; yellow/grid pattern = weakest appreciation.

Figure 3 presents the use and appreciation per app component. For readers interested in intervention development, a joint display of use and appreciation per app component (including frequency data and qualitative themes) can be found in Supplementary File 3. Audio-exercises were particularly appreciated, with adequate and pleasant tempo and duration, tone of voice and content. Similarly, light of the day and push notifications were highly appreciated (i.e. representing very brief inspirational content); many participants continued to engage with push notifications long after the intervention period was over (*‘even though I don’t open the app anymore, I really enjoy reading those notifications*’ [D070]). As shown in Figure 3, the light of the day had a relatively low use despite high appreciation, and participants reported incorporating this into their lives in various ways without using the app (‘*[the light of the day] really stood out to me (…) I wrote it down on notes and put them in a large glass vase*’ [D090]). In contrast, the mood tracker had a similar low use, but also relatively low appreciation. Many participants found it too intensive to track their emotions or did not see the added value. Overall, the results indicate that use and appreciation of app components corresponded with their intended design. For example, info and links was considered supportive but low-priority, and the optional peer experiences were considered highly valuable for some participants but too confronting for others.

### Impact

#### Mental health outcomes

Participants reported improved mental health outcomes after using the app (see Table 3). Qualitative mental health themes were experiencing rest (‘*[the app] gave me more rest*’ [D039]) and positive emotions (*‘it really made me feel more joy*’ [D028])*.* Quantitative data showed a significant reduction in anxiety (*t*(35) = 2.66, *p* = .01) (no significant changes in depression or mental wellbeing) from pre- to post-intervention. There were no qualitative themes and no pre-post differences in social isolation (PROMIS-SI; (*t*(35) = -0.99, *p* = .33) and resilience (BCRS; (*t*(35) = 0.27, *p* = .79), therefore no mixed themes are reported in Table 3. No negative or adverse outcomes were reported by participants. No differences on demographic and illness characteristics (e.g. gender, education, type of diagnosis) were found between people whose mental health improved versus whose did not, except that participants whose (cancer-related) resilience improved from pre- to post-intervention were more recently diagnosed than others (*t*(34) = 1.29, *p *< 0.01). Changes in mental health outcomes were not significantly correlated with higher app appreciation or with higher app use (number of modules or audio-guided exercises used, total use or self-reported time spent on app). Except when comparing user and non-users per additional module used, people who used all 6 modules improved significantly more on self-reassurance than those who used 5 or less modules (*t*(32) = 2.18, *p *= .04).

**Table 3.** Joint display of experienced mental health benefits after using Compas-Y.

Mixed theme	Qualitative themes	Illustrative quotes	Quantitative findings				Mixed interpretation
Mental health	Experiencing rest	‘When I am using it, (…) it offers some sort of relaxation’ [D092]	Variable	Pre-mean	Post-mean	Change	Participants reported that the app helped them to experience rest and positive emotions. These themes are akin to anxiety (e.g. ‘I can sit at ease and feel relaxed’) and depression (e.g. ‘I feel cheerful’) on the HADS scale. Indeed, there is a sign. Reduction in anxiety (but not depression or well-being) from pre- to post-intervention. The app seems to help participants to feel more at ease and less anxious.
			Anxiety (HADS)	8.6 (4.0)	6.0 (3.0)	*t*(35) = 2.66, *p* = .01* (*d* = .44)	
	Experiencing positive emotions	‘[the app] helps to feel more positive through it all’ [D092]					
			Depression (HADS)	6.1 (2.0)	5.6 (3.6)	*t*(35) = 1.85, *p* = .53	
			Mental well-being (MHC-SF)	3.2 (1.0)	3.1 (.7)	*t*(36) = 0.55, *p* = .59	

Note: The results on the mental well-being (MHC-SF) subscales were similar to the overall score and are not reported for ease of interpretation.

* Significant at the level of *α* < .05.

### Process outcomes

Participants reported gaining self-awareness, recognition and support due to using the app (e.g. ‘*Physically you have to endure so much, and then mentally this app is a huge support*’ [D050] and ‘*[the app] helps you to reflect on everything that is going on*’ [D004]) (see Table 4 for an overview of process outcomes). Since there were no qualitative themes and no pre-post differences on fears of giving compassion (FOCS; *t*(35) = -0.90, *p* = .38) or fears of receiving compassion (FOCS; *t*(35) = 0.32, *p* = .75), fears of compassion is not part of the mixed themes in Table 4. Participants also reported improved self-compassion and self-criticism after using the app. Qualitative self-compassion themes include having learned to be mild to yourself (‘*[the app gave me] tools to be more kind to myself*’ [VD075]) and to set boundaries (‘*[after using module 5] I kindly said: you don’t have to visit me 3 times a week*’ [D037]). No significant pre-post changes on self-compassion were found, while significant pre-post differences were found for self-blame (*t*(34) = 2.20, *p* = .04) and inadequate self (*t*(35) = 2.35, *p* = .03). When comparing people whose process outcomes improved after using the app versus whose did not, we found that people whose self-blame improved were more recently diagnosed (*t*(33) = 0.993, *p *= .02). No other differences in demographic and illness characteristics (e.g. gender, education, type of diagnosis) were found. Changes in quantitative outcome variables were not significantly correlated with higher app appreciation or with higher app use (number of modules or audio-guided exercises used, total use or self-reported time spent on app).

**Table 4.** Joint display of experienced process benefits after using Compas-Y.

Mixed theme	Qualitative themes	Illustrative quotes	Quantitative findings				Mixed interpretation
Self-awareness, recognition and support	Self-awareness and self-reflection	‘You recognize more what is going on in your mind’ [D075]	50% (totally) agreed with that the app helped them to gain ‘insight into emotions’ (TWEETS, *n *= 34).				Participants described that the app helped them to be more aware of their mental and emotional experiences, and to realise that they are not alone or exceptional for having these experiences. The app also provided a sense of support throughout difficult times.
	Recognition and normalisation	‘The things you go through and experience, that they are actually quite universal’ [D070]					
	Support	‘It's like you have a friend on your phone, who already went through this, or who knows what you need’ [D060]					
Self-compassion and self-criticism	Insight into importance of self-compassion	‘[I learned] that self-compassion is important, a good skill to develop’ [D070]	61.8% (totally) agreed with that the app ‘made it easier to be self-compassionate’ and 67.6% (totally) agreed with that the app ‘motivated me to be self-compassionate’ (TWEETS, *n *= 34).				Participants reported to find recognition and confirmation of their self-compassion, which aligns with the already very high baseline score of self-compassion. The high baseline may explain the lack of a pre-post difference (ceiling effect) on self-compassion. Nevertheless, participants described numerous experiences of increased self-compassion after using the app (being mild to yourself, asking for help, etc.), and there may be a difference in how these benefits are self-described vs. how they are phrased in survey items.
	Insight into own self-compassion	‘More insight into my compassionate self’ [VD093]					
			Variable	Pre-IV mean	Post-IV mean	Change	
	Being mild to yourself	‘I still do that [being mild to myself], (…) I say to myself: you happen to be in this situation’ [D053]	Self-compassion (SCS-SF)	4.9 (1.0)	5.0 (.8)	*t*(36) = −1.09, *p = *.28	
	Taking care of yourself	‘What the app really offers, is putting yourself on no.1. Not in the sense that everything revolves around you, but more like: what is a healthy way to take care of myself’ [D088]	*Reassured self (FSCSRS-SF)*	14.9 (3.2)	13.9 (3.4)	*t*(35) = 2.88, *p = *.08	

Mixed theme	Qualitative themes	Illustrative quotes	Quantitative findings				Mixed interpretation
(Self-compassion and self-criticism)	Being less harsh/strict with yourself	‘Through the app I learned to say: I don’t have to be harsh with myself’ [D022]	*Inadequate self (FSCSRS-SF)*	6.5 (4.2)	5.6 (4.0)	*t*(35) = 2.35, *p = *.03* (*d *= .39)	Further reported that the app enabled them to be less harsh or strict with themselves. Indeed, there are sign. pre-post reductions on subscales inadequate self (self-criticism) and self-blame (coping), despite the already low baseline scores.
	Asking for help	‘I would have done it all myself, I have learned to delegate’ [D022]	*Self-blame (CERQ)*	3.5 (2.0)	3.0 (1.5)	*t*(34) = 2.20, *p* = .04* (*d* = .37)	
	Setting boundaries (social and physical)	‘I stand up for myself more, I don’t have to keep everybody happy’ [D040]					
			
Emotion regulation and coping	Creating moments of rest	‘Some days you really need rest, and then there are things in the app that remind you of that’ [D039]	Variable (CERQ)	Pre-IV mean	Post-IV mean	Change	Participants reported experiences of increased awareness and acceptance of emotions and having more strategies to handle emotions. Participants further reported being more accepting of their situation, and actively creating moments of rest and positivity. Yet there were no sign. pre-post differences on any of the coping strategies, including acceptance and positive refocusing. This divergence may be due to very high baseline scores (ceiling effect), as well as a difference in how process
			*Positive reappraisal*	6.9 (2.1)	6.4 (2.1)	*t*(34) = 1.27, *p* = .21	
	Savoring positive experiences	‘Try to reflect every day on what went well, what was nice about the day’ [D004]	*Positive refocusing*	6.3 (1.7)	6.2 (1.5)	*t*(34) = 0.44, *p* = .67	
	Acceptance of changes	‘because of the app, it is easier to accept that you’re changing [due to (…)] so many different medications and treatments’ [D093]	*Acceptance*	8.2 (1.5)	7.8 (1.6)	*t*(34) = 1.58, *p* = .12	
			*Putting into perspective*	5.2 (2.0)	5.3 (2.2)	*t*(34) = -0.26, *p* = .78	

Mixed theme	Qualitative themes	Illustrative quotes	Quantitative findings				Mixed interpretation
(Emotion regulation and coping)	Being aware of emotions	‘I started to become aware of all the thoughts and feelings I have’ [vD089]	*Planning*	5.8 (2.0)	5.7 (1.9)	*t*(34) = 0.50, *p* = .62	Benefits are self-described vs. how they are phrased in survey items.
	Accepting emotions	‘There are moments when I’m afraid, but I also know it's part of the process and I accept that’ [D022]	*Catastrophising*	3.9 (1.3)	5.2 (2.0)	*t*(34) = 0.72, *p* = .48	
			*Rumination*	5.8 (1.5)	5.4 (1.4)	*t*(34) = 1.71, *p* = .10	
	Handling emotions	‘The feeling [anger] is part of it, but then you can flip the switch’ [D092]	*Other-blame*	2.5 (1.4)	2.4 (1.4)	*t*(34) = 1.15, *p* = .26	

Note: The results on the self-compassion positive and negative composite scores (SCS-SF) were similar to the overall score and are not reported for ease of interpretation.

Note: The results on the self-compassion positive and negative composite scores (SCS-SF) were similar to the overall score and are not reported for ease of interpretation.

* = significant at the level of *α* < .05.

## Discussion

This study was the first to evaluate a mobile compassion training for people with cancer (*Compas-Y*) and one of the first evaluations of a technology-enabled compassion training in general. Using a convergent mixed methods design, this pilot study with 71 people with cancer sought to examine the use, appreciation and impact of *Compas-Y*. The results indicate that the intervention is highly appreciated and helped users to improve aspects of their mental health, while app use was relatively low. Furthermore, no negative or adverse effects were reported. Given the rise of eHealth and the way it is counted upon to contribute to challenges of capacity and accessibility in health care,^1,56,57^ this study contributes a concrete example of how people with cancer can be offered low-threshold support.

Intervention appreciation was high for nearly all intervention aspects, including form aspects such as language and lay-out, virtually all of the intervention content and most of the supportive functionalities. Module themes such as the three emotion systems and fostering positivity were especially appreciated for being relevant and adapted to the context of cancer. In other compassion-and mindfulness-based interventions, components tailored to the illness context are often highlighted as particularly helpful by participants, while in untailored interventions illness-specific content is oftentimes suggested as a point for improvement.^21,58^ Nevertheless, it is unclear whether tailored interventions are more used, appreciated, or impactful than untailored ones, and comparative studies are needed. In the current evaluation study, the high appreciation and the few suggestions for improvement suggest that user wishes, needs and experiences were adequately assessed and implemented, underlining the importance of involving end-users in the development and tailoring process. Simultaneously, the theory and evidence-based requirements for the intervention were met, encompassing most essential components of CMT.^19^ Thus, the results confirm a successful top-down bottom-up co-design process (see Ref.^18^).

Similar to most eHealth and mHealth interventions,^59,60^ app use dropped from 97% for the first to 27% of participants for the last module, with a use of 40–65% for the supportive functionalities such as the mood tracker. Reasons for (temporary) disengagement emerging from the interviews and reasons for study drop-out included not having enough energy or being too sick, which seems to be a commonly reported reason for drop-out in cancer-related eHealth interventions.^61,62^ Importantly, participants also described implementing exercises outside of the app, which may be a reason why we did not find many dose-response relations between app use and outcome measures. Furthermore, having gained sufficient support from the app and being mild to yourself were reasons for disengagement. Thus, low use was not necessarily conflicting with intervention aims. Indeed, a decrease in distress (and consequently a lower support need) may cause lower adherence,^63^ and presuming dose-response relations may not account for users’ personal goals.^64^ As disengagement can be seen as a typical feature of eHealth, it is important to gain further insights into types of and reasons for adherence and attrition.^60^ Our pilot study offered little indication for significant differences in frequency of use based on demographic or illness variables, indicating that the app is suitable for a wide range of people. Future research could gain more in-depth insights into different use patterns and user motivations, for example with *N*-of-1 trials.^29^

Participants reported improved mental health outcomes after using the app, as evidenced by a statistically significant reduction in anxiety (*t*(35) = 2.66, *p* = .01) and qualitative themes of experiencing more rest and more positive emotions. These reported benefits are akin to benefits experienced after participating in face-to-face compassion-based interventions.^21^ Participants further report a rich plethora of process benefits, including gained self-awareness, recognition and support; improved self-compassion and reduced self-criticism; and increased emotion regulation and coping skills. Most of these process benefits are primarily supported by qualitative themes, except for self-criticism (with significant reductions in inadequate self and self-blame). It should be noted that participants generally reported relatively high levels of mental health before using the app, thus a ceiling effect may have prevented significant improvement.^65^ Furthermore, it seems that there is a discrepancy between the way people describe their daily life experiences of self-compassion and self-criticism, and the way that these concepts are typically measured by self-report surveys.^12^ Our review of compassion-based interventions for people with long-term physical conditions showed minimal overlap between qualitative themes of experienced benefits and quantitative outcome measures.^21^ In the current study we attempted to bridge this gap with a convergent mixed-method approach. Yet it may be that self-report instruments are not measuring the full range of intervention benefits that participants experience, and multimodal assessment of self-compassion remains vital to enable to learn from convergence and divergence in outcomes (see Ref.^66^).

Interestingly, results showed that people with higher logged app use as well as higher app appreciation were more likely to already have some familiarity with self-compassion or mindfulness. This could indicate that prior familiarity may make it easier to engage with the intervention, especially in a context of having little energy or focus. Compassion training can be challenging, as it requires some degree of acknowledging or engaging with suffering, which may elicit its own fears, blocks and resistances.^67^ Nevertheless, the high ratings of intervention content, information modalities and ease of language do suggest that *Compas-Y* is an accessible intervention. In this study we particularly reached people already high in self-compassion, despite that self-compassion was not centralised in recruitment information (although nurses may have inadvertently selected people for whom they deemed self-compassion fitting). It is conceivable that people high in self-compassion are most interested in the app, or perhaps less likely to discount compassion as overly soft or woolly.^68^ Alternatively, it may be that the situation of receiving a cancer diagnosis particularly requires a self-compassionate response (i.e. participants have high state self-compassion). Research indicates that being diagnosed with cancer can elicit both self-compassionate and self-critical responses,^12,69^ and this complex dynamic likely depends on temporal, contextual and individual factors.^70^ Longitudinal methods could shed light on some of the moment-to-moment variation in self-compassion and self-criticism while using the intervention.^71^ Furthermore, alternative recruitment strategies and their impact could be investigated, such as explicitly recruiting people who identify as being too self-critical or low in self-compassion.^23^

Lastly, in the evaluation of individual app components, it appears that very brief inspirational content such as compassionate push notifications and the light of the day exercise are particularly appreciated and implemented into daily life. Such ‘popcorn style’ content may be the most feasible in the context of having little energy or focus, and/or most congruent with what people have come to expect from using a mobile device.^72^ In contrast, regular use of the mood tracker (i.e. tracking (daily) mood on three scales) was found too intensive by participants. Similar to other apps there seems to be a need to offer users more preparation for tracking by explaining the use and added value.^73^ Importantly, the mood tracker in the app was designed not only for self-tracking, but also as a personalised entry point into the intervention, with text-based supportive suggestions offered based on the currently reported mood. This constituted an addition to the tunnelled training structure, and future intervention designs could explore current reports of mood and/or self-compassion as a primary gateway for an intervention, that is, offering psycho-education and exercises in response to a user's current state. Despite these considerations, the training modules were highly appreciated by those who used them, offering a source of support and preparation particularly shortly after diagnosis. Given the large variety in symptom and fatigue trajectories in people with cancer,^74–76^ it may be a matter of making sure that the intervention is available at the right time for individual users (e.g. by (re-)offering the intervention at different timepoints). For further evaluation of Compas-Y, a next step would be to conduct a controlled evaluation study with initial minor modifications (e.g. increased guidance towards the mood tracker) and a wider target audience (e.g. people with low mental well-being), after which potential essential or redundant intervention components may be further clarified.

This study was strengthened by its mixed methods approach, which is vital for the comprehensive evaluation of digital interventions.^33^ This included objective and subjective data, and integrative data analysis, something that is often missing even in mixed methods studies.^77–79^ Yet the results should be interpreted in light of some limitations. First, the correlational nature of this pilot study and lack of control group preclude conclusions regarding causality of experienced benefits. Given that most participants were recently diagnosed with cancer, it can be assumed that they went through many changes irrespective of the intervention. Therefore, it is unknown to what extent quantitative pre-post differences can be attributed to the intervention. Second, most participants were women, with a Dutch cultural heritage and in a committed relationship. Since gender role socialisation and ethnic minority status may impact experiences of self-compassion^80,81^), the generalisability of these results to all people with cancer is unclear. Third, while non-adherence was similar to other eHealth studies, the pre-post results were skewed towards participants who had the energy, focus or motivation to continue participating in the study despite their illness (while participants were invited to complete post-test surveys regardless of app (non)use, people who completed surveys had significantly higher app use). Fourth, due to the scope of this article we only conducted basic frequency analysis of log-data, and follow-up analysis coupled with ESM data is planned. Further research could evaluate the use, appreciation and impact of the intervention in a (quasi-)experimental design, as well as increase efforts towards specifically addressing underrepresented populations, for example, with culturally sensitive recruitment strategies.^82,83^

In summary, this mixed methods pilot study presented the first evaluation of a mobile self-compassion intervention for people with newly diagnosed cancer, examining its use, appreciation and impact. Key implications are presented in the textbox below.

**Table:** 

**Key clinical, theoretical and practical implications** CMT seems to be well suited to the lived experiences of people with newly diagnosed cancer Compas-Y appears to support mental health shortly after diagnosis, although controlled research and further implementation is needed Future efforts should focus on how to include more diverse participants with a high support need App use is not indicative for how much a user is actively working with intervention content Participants differed in their preferences for free vs. tunnelled content, experimental research is needed to examine variations of interventions

Results indicated that the intervention was highly appreciated, with all intervention topics considered relevant and a source of support shortly after diagnosis. This underscores the success of co-designing this intervention together with people with cancer and oncology nurses. Similar to other eHealth studies, non-adherence was high, as only 27% of participants used all of the six training modules. Yet reasons for disengagement were not always conflicting with intervention goals, for instance when participants implemented exercises outside of the app. Experienced benefits of the intervention included improved mental health, and process benefits of gained self-awareness, recognition and support; improved self-compassion and self-criticism; and increased emotion regulation and coping skills. Further research is needed to examine variations in use patterns and use of different app components, fluctuations in self-compassion throughout intervention use and recruitment and implementation strategies that address a diversity of people with cancer at the right time in their illness trajectory. This pilot study offered a promising first evaluation of *Compas-Y*, demonstrating the suitability of a unique co-designed version of CMT to the modality of mobile technology and the context of a recent cancer diagnosis.

## Supplemental Material

sj-docx-2-dhj-10.1177_20552076231205272 - Supplemental material for Compas-Y: A mixed methods pilot evaluation of a mobile self-compassion training for people with newly diagnosed cancer Click here for additional data file. Supplemental material, sj-docx-2-dhj-10.1177_20552076231205272 for Compas-Y: A mixed methods pilot evaluation of a mobile self-compassion training for people with newly diagnosed cancer by Judith Austin, Maya J Schroevers, Jelle Van Dijk, Robbert Sanderman, Elin Børøsund, A Machteld N Wymenga, Ernst T Bohlmeijer and Constance H.C. Drossaert in DIGITAL HEALTH

sj-docx-3-dhj-10.1177_20552076231205272 - Supplemental material for Compas-Y: A mixed methods pilot evaluation of a mobile self-compassion training for people with newly diagnosed cancer Click here for additional data file. Supplemental material, sj-docx-3-dhj-10.1177_20552076231205272 for Compas-Y: A mixed methods pilot evaluation of a mobile self-compassion training for people with newly diagnosed cancer by Judith Austin, Maya J Schroevers, Jelle Van Dijk, Robbert Sanderman, Elin Børøsund, A Machteld N Wymenga, Ernst T Bohlmeijer and Constance H.C. Drossaert in DIGITAL HEALTH

sj-docx-4-dhj-10.1177_20552076231205272 - Supplemental material for Compas-Y: A mixed methods pilot evaluation of a mobile self-compassion training for people with newly diagnosed cancer Click here for additional data file. Supplemental material, sj-docx-4-dhj-10.1177_20552076231205272 for Compas-Y: A mixed methods pilot evaluation of a mobile self-compassion training for people with newly diagnosed cancer by Judith Austin, Maya J Schroevers, Jelle Van Dijk, Robbert Sanderman, Elin Børøsund, A Machteld N Wymenga, Ernst T Bohlmeijer and Constance H.C. Drossaert in DIGITAL HEALTH
