# Supplementary material for: Compas-Y: A mixed methods pilot evaluation of a mobile self-compassion training for people with newly diagnosed cancer
Source: Digit Health. 2023 Oct 19;9:20552076231205272. doi: 10.1177/20552076231205272 (PMC10588427; doi:10.1177/20552076231205272)
Supplement: sj-docx-2-dhj-10.1177_20552076231205272 - Supplemental material for Compas-Y: A mixed methods pilot evaluation of a mobile self-compassion training for people with newly diagnosed cancer [file sj-docx-2-dhj-10.1177_20552076231205272.docx]

## Supplementary File 1

**Brief Cancer-related Resilience Scale**

Q1. I tend to bounce back quickly after a difficult phase of my illness or treatment

Q2. I have a hard time making it through when I experience side effects, pain or limitations

Q3. It does not take me long to recover from a difficult phase of my treatment

Q4. It is hard for me to snap back after a “bad news” consult

Q5. I usually come through setbacks related to cancer with little trouble

Q6. I need a long time to get over the fact that I have cancer

Adaptation of the Brief Resilience Scale ^1^

1. Smith BW, Dalen J, Wiggins K, et al. The brief resilience scale: Assessing the ability to bounce back. *International Journal of Behavioral Medicine* 2008; 15: 194-200. DOI: 10.1080/10705500802222972.
