# Supplementary material for: Compas-Y: A mixed methods pilot evaluation of a mobile self-compassion training for people with newly diagnosed cancer
Source: Digit Health. 2023 Oct 19;9:20552076231205272. doi: 10.1177/20552076231205272 (PMC10588427; doi:10.1177/20552076231205272)
Supplement: sj-docx-3-dhj-10.1177_20552076231205272 - Supplemental material for Compas-Y: A mixed methods pilot evaluation of a mobile self-compassion training for people with newly diagnosed cancer [file sj-docx-3-dhj-10.1177_20552076231205272.docx]

## Supplementary File 2

*Comparison of participants who created app account with norm scores on psychosocial measures*

| Item | Mean (SD) | Mean general/student samples | Mean people  with cancer  samples | Difference between samples |
| --- | --- | --- | --- | --- |
| Self-compassion | 57.79 (12.15) | 48.12 |  | *t*(68)=6.61, *p*<.001 |
|  |  |  | 39.70 | *t(68)*=12.37, *p*<.001 |
| Mental well-being | 3.15 (.94) | 2.98 |  | *t*(68)=1.49, *p*=.14 |
| Resilience* | 3.41 (.64) | 3.53 |  | *t*(68) =-1.55, *p*=.13 |
| Self-criticism |  |  |  |  |
| *Inadequate self* | 6.68 (3.99) | 8.66 |  | *t*(68) =-4.12, *p*<.001 |
| *Reassured self* | 14.35 (3.39) | 13.29 |  | *t*(68) =-2.59, *p*=0.01 |
| Depression | 6.37 (3.70) | 3.68 |  | *t*(66)=5.96, *p*<.001 |
|  |  |  | 6.40 | *t*(66)=-.06, *p*<.95 |
| Anxiety | 8.13 (4.14) | 6.14 |  | *t*(66)=3.94, *p*<.001 |
|  |  |  | 7.20 | *t(66)=1.85, p<.07* |
| Coping |  |  |  |  |
| *Acceptance* | 7.90 (1.64) | 5.49 |  | *t*(68)=12.22, *p*<.001 |
| *Rumination* | 5.70 (1.71) | 4.98 |  | *t* (68)=3.48, *p*<.001 |
| *Positive reappraisal* | 6.70(2.16) | 6.23 |  | *t* (68)=1.79, *p* =.08 |
| *Self-blame* | 3.46(1.80) | 4.13 |  | *t* (69)=-3.03, *p*<.01 |
| *Positive refocusing* | 6.42 (1.83) | 4.54 |  | *t* (68)=8.52, *p*<.001 |
| *Other-blame* | 2.33(1.09) | 3.10 |  | *t* (68)=-5.82, *p*<.001 |
| *Planning* | 5.89 (2.10) | 6.05 |  | *t* (68)=-.60, *p*=.55 |
| *Catastrophizing* | 3.79 (1.53) | 3.30 |  | *t* (68)=2.70, *p*<.01 |
| *Putting into perspective* | 5.28 (1.98) | 5.85 |  | *t* (68)=-2.41, *p*=.02 |

References for norm scores: Raes et al. (2011) & Zhu et al. (2019; SCS-SF self-compassion); Lamers et a. (2011; MHC-SF mental well-being); Smith et al. (2008; BRS resilience, based on the original/unadapted scale); Sommers-Spijkerman et al. (2018; FSCRS-SF self-criticism); Crawford et al. (2001) & Singer et al. (2009; HADS depression and anxiety); Garnefski et al. (2006, CERQ-18 coping). No suitable norms were found for social isolation (PROMIS-SF) and fears of compassion (FOCS).

^~~1-8~~^

## References

1. Raes F, Pommier E, Neff KD, et al. Construction and factorial validation of a short form of the Self-Compassion Scale. *Clinical Psychology & Psychotherapy* 2011; 18: 250-255. <https://doi.org/10.1002/cpp.702>. DOI: <https://doi.org/10.1002/cpp.702>.

2. Lamers SM, Westerhof GJ, Bohlmeijer ET, et al. Evaluating the psychometric properties of the Mental Health Continuum-Short Form (MHC-SF). *Journal of clinical psychology* 2011; 67: 99-110. DOI: 10.1002/jclp.20741.

3. Smith BW, Dalen J, Wiggins K, et al. The brief resilience scale: Assessing the ability to bounce back. *International Journal of Behavioral Medicine* 2008; 15: 194-200. DOI: 10.1080/10705500802222972.

4. Sommers-Spijkerman M, Trompetter H, ten Klooster P, et al. Development and validation of the forms of Self-Criticizing/Attacking and Self-Reassuring Scale—Short Form. *Psychological Assessment* 2018; 30: 729-743. DOI: 10.1037/pas0000514.

5. Crawford JR, Henry JD, Crombie C, et al. Normative data for the HADS from a large non-clinical sample. *Br J Clin Psychol* 2001; 40: 429-434. DOI: 10.1348/014466501163904.

6. Garnefski N and Kraaij V. Cognitive emotion regulation questionnaire – development of a short 18-item version (CERQ-short). *Personality and Individual Differences* 2006; 41: 1045-1053. DOI: <https://doi.org/10.1016/j.paid.2006.04.010>.

7. Singer S, Kuhnt S, Götze H, et al. Hospital anxiety and depression scale cutoff scores for cancer patients in acute care. *Br J Cancer* 2009; 100: 908-912. 20090224. DOI: 10.1038/sj.bjc.6604952.

8. Zhu L, Yao J, Wang J, et al. The predictive role of self-compassion in cancer patients' symptoms of depression, anxiety, and fatigue: A longitudinal study. *Psycho-Oncology* 2019; 28: 1918-1925. Article. DOI: 10.1002/pon.5174.
