# Supplementary material for: Compas-Y: A mixed methods pilot evaluation of a mobile self-compassion training for people with newly diagnosed cancer
Source: Digit Health. 2023 Oct 19;9:20552076231205272. doi: 10.1177/20552076231205272 (PMC10588427; doi:10.1177/20552076231205272)
Supplement: sj-docx-4-dhj-10.1177_20552076231205272 - Supplemental material for Compas-Y: A mixed methods pilot evaluation of a mobile self-compassion training for people with newly diagnosed cancer [file sj-docx-4-dhj-10.1177_20552076231205272.docx]

## Supplementary File 3

Formative joint display of app use and appreciation per app component

| **App component** | **Use: qualitative findings** | **Use: quantitative findings** | **Appreciation: qualitative findings** | **Appreciation: quantitative findings** | - **Mixed method interpretation** |
| --- | --- | --- | --- | --- | --- |
| **Audio-guided exercises** | *Characteristics of use:* Save for specific moments Sticking with one voice  *Reasons for non-use:*  Use other familiar meditation exercises instead | 68% played >1 audio-exercise, 30% played audio-exercises repeatedly (4-18 times) (*n*=71). | Supportive and calming Clear and pleasant content & tone of voice Good duration and tempo Good to have choices in voices and text | Most often self-described as favorite aspect of app (5/38 answers). | Audio-guided exercises are perceived as supportive, with an appropriate tone of voice, duration and tempo. No negative evaluations or app-specific reasons for non-use are reported. |
| **Reflective and applied exercises** | *Characteristics of use:* Reflection exercises in thoughts  Save exercises for later | x | Pleasant and valuable Helpful reflective questions  Typing in the app can be difficult/of little added value | 47.1% (*n*=34) appreciated the exercises mostly or completely.  Most users wrote either in the app (35.1%) or on paper (32.4%; *n*=37), and were satisfied with their choice (81.5%, of *n*=27). | The exercises are considered pleasant and helpful, while not fitting for everyone.  Writing in the app was difficult for some, yet this was still the preferred way of doing exercises overall. |
| **Peer experiences** | *Characteristics of use:* Either use or consciously avoid | 55 (77.5%) looked >1 through peer experiences, 16 (22.5%) did not open any (*n*=71). | Pleasant and anonymous Offers recognition Offers different perspectives Can be confronting Not fitting for me | Second most often self-described as favorite aspect of app (4/38 answers). | Peer experiences are used and appreciated by some, and consciously avoided by others. Participants either find them helpful (pleasant, offering recognition, offering different perspectives in an anonymous way) or confronting and not fitting. This is consistent with the intention and design of this component as optional. |
| **Mood tracker** | *Characteristics of use:*  Mostly in the beginning/first weeks  *Reasons for non-use*:  Not experiencing need to track No time or headspace Not enough variation in emotions Tracking makes life less normal Did not notice functionality | 8.8% (mostly) daily use (*n*=34); 51.4% of users  tracked >1 mood (*n*=71)  43.7% of users viewed >1 mood feedback (*n*=71) | Clear and pleasant  Good to reflect on emotions Hard to interpret Tracking does not offer much added value  *Suggestions for improvement:*  Explain the mood tracker better Combine experience sampling methods (ESM) with mood tracker | 17.6% (*n*=34) mostly or completely appreciated the mood tracker. | While helpful and pleasant for some, tracking mood is too intensive or not relevant for many. The functionality is also not noticeable for everyone. Some track a few times early on for initial insight. The intended aim of connecting to the modules by giving personalized feedback is not mentioned by participants and could be more clear. Of note, these results seem confounded with the ESM study. |
| **Light of the day** | *Characteristics of use:* Track light of the day elsewhere inspired by app (on paper, social media) Integrate light of the day exercise in mindset (no tracking) | 17,6% self-reported (mostly) daily use (*n*=34); 44.3% of users logged >1 light (*n*=71) | Helpful and insightful Pleasant and valuable Fitting to the illness context | Second most often self-described as favorite aspect of app (4/38 answers).  41.2% (*n*=34) mostly or completely appreciated the light of the day. | The exercise light of the day is considered pleasant, insightful and fitting to the illness context, and inspires full integration into daily life (beyond the frequency of in-app tracking). It may not be for everyone, since only 41.2% report to fully appreciate it, yet all feedback is exclusively positive. |
| **My favourites** | *Reasons for use:*  To find favorite exercises again To return to skipped content  *Reasons for non-use:*  Did not notice functionality Already integrated exercises Prefer to repeat modules in their entirety | 5.9% self-reported (mostly) daily use (*n*=34); 40.0% of users saved >1 exercise (*n*=71) | Helpful to find and return to components Necessary, without it components are hard to find | 5.9% (*n*=34) mostly or completely appreciated my favorites. | Some find my favorites helpful to return to favorite or skipped content, yet many did not notice or appreciate it. No negative evaluations of the functionality itself are given, and the main issue may be to make it more noticeable for those who want to use it. |
| **Info & links** | *Reasons to use:*  Getting reliable information Knowing content for when need arises  *Reasons for non-use:* Low need for more information Found information elsewhere/earlier Did not notice functionality | 5 (14.7%) report looking at most of the info & links and 15 (44.1%) partly (*n*=34).  46 (64.8%) opened Info & Links >1 time (*n*=71). | Promotes trust in the app Offers reliable information Can be confronting Not a priority | 38.2% (*n*=34) mostly or completely appreciated info & links. | Info & links is appreciated for its reliable information, but is not considered a priority and is used infrequently. This is in line with its aim as a background information source. Improved guidance towards this functionality could make it more noticeable. |
| **Push notifications** | *Characteristics of use:* Continuing to read notifications after discontinued app use Notifications as inspiration only Notifications as reminder for app use  Saving notifications outside of the app | X | Nice and pleasant Supportive and insightful  Good reminder for app  Not fitting for me (content/time/frequency) Confusing to receive via e-mail (iOS)  *Suggestions for improvement:*  Include explicit reminders for exercises Send notifications via phone (iOS) | Most often self-described as favorite aspect of app (5/38 answers).  47.1% (of *n*=34) mostly or completely appreciated push notifications. | Notifications are considered pleasant and supportive. For some notifications are used as a reminder, for others they are stand-alone (particularly for iOS users who received them by e-mail). Some save the content of notifications externally, and many continue to engage with them after the intervention period has passed. |
